# Editorial: Electrochemical Sensors and Biosensors in Medical and Pharmaceutical Bioanalysis

**DOI:** 10.3389/fbioe.2020.00533

**Published:** 2020-05-29

**Authors:** Constantin Apetrei, María Luz Rodriguez-Mendez, Mihaela Badea, Cecilia Cristea

**Affiliations:** ^1^Department of Chemistry, Physics and Environment, Faculty of Science and Environment, Dunarea de Jos University of Galati, Galati, Romania; ^2^Group UVASens, Department Inorganic Chemistry, Engineers School, University of Valladolid, Valladolid, Spain; ^3^Faculty of Medicine, Transilvania University of Brasov, Brasov, Romania; ^4^Analytical Chemistry Department, Faculty of Pharmacy, Iuliu Hatieganu University of Medicine and Pharmacy, Cluj-Napoca, Romania

**Keywords:** paper-based electrochemical device, nanotoxicity, blood coagulation, nucleic acid, lung cancer biomarker, D-phenylalanine

This Research Topic collects different contributions in the emerging field of bioanalysis, highlighting the most relevant advances reported in the literature as well as some original research studies in medical and pharmaceutical bioanalysis.

The first article of this Topic (Antonacci et al.), describes in a detailed review state of the art the paper-based electrochemical devices for pharmaceutical field. In the last few decades, scientific research has been trying to offer valid and reliable solutions to replace or support conventional techniques, in order to facilitate drug development procedures. These innovative approaches may have extremely positive effects in the production chain, supplying fast and cost-effective quality and safety tests on active pharmaceutical ingredients and their excipients. In this context, the exploitation of electrochemical paper-based analytical devices is still in its infancy, but particularly promising for its fascinating properties in the detection of active pharmaceutical ingredients and excipients in tablets, capsules, suppositories, and injections, as well as for pharmacokinetic bioanalysis in real samples.

In a comprehensive review, Shinde et al. attempts to provide a detailed information on the recent advancements made in development of bioassay models and systems for assessing the nanotoxicology. Bioelectrochemical techniques, with the recent advancements in the microelectronics, proved to be capable of providing non-invasive measurement of the nanotoxicity effects both at single cellular and multicellular levels. This review highlights the multiple bioassay models evolved for toxicological studies. Emphasize on multiple mechanisms involved in the cell toxicity and electrochemical probing of the biological interactions, revealing the cytotoxicity were also provided. Limitations in the existing electrochemical techniques and opportunities for the future research focusing the advancement in single molecular and whole cell bioassay has been discussed.

The last review of this Topic (Aria et al.) is related to the technology advancements in blood coagulation measurements for diagnosis of bleeding disorders. In recent years, blood coagulation monitoring has become crucial to diagnosing causes of hemorrhages, developing anticoagulant drugs, assessing bleeding risk in extensive surgery procedures and dialysis, and investigating the efficacy of hemostatic therapies. In this regard, advanced technologies such as microfluidics, fluorescent microscopy, electrochemical sensing, and photoacoustic detection, and micro/nano electromechanical systems have been employed to develop highly accurate, robust, and cost- effective point of care devices. This devices measuring electrochemical, optical, and mechanical parameters of clotting blood. The working principle of blood coagulation monitoring, physical, and sensing parameters in different technologies have been discussed. On the other hand, it has been discussed the recent progress in developing nanomaterials for blood coagulation detection and treatments. Moreover, commercial products, future trends/challenges in blood coagulation monitoring including novel anticoagulant therapies, multiplexed sensing platforms, and the application of artificial intelligence in diagnosis and monitoring have been included.

An original study (Wang et al.) describes a novel method for simultaneous nucleic acids detection and elimination of carryover contamination with nanoparticles-based biosensor- and Antarctic thermal sensitive uracil-DNA-glycosylase-supplemented polymerase spiral reaction. The technique merges enzymatic digestion of carryover contaminants and isothermal nucleic acid amplification technique for simultaneous detection of nucleic acid sequences and elimination of carryover contamination. In particular, nucleic acids amplification and elimination of carryover contamination are conducted in a single pot, and thus, the use of a closed-tube reaction can remove undesired results due to carryover contamination. For demonstration purpose, *Klebsiella pneumoniae* is employed as the model to demonstrate the usability of the assay. The amplification products were detectable from as little as 100 fg of genomic DNAs, and from ~550 CFU in 1 mL of spiked sputum samples. All *K. pneumoniae* strains examined were positive for NB-ATSU-PSR detection, and all non-*K. pneumoniae* strains tested were negative for the NB-ATSU-PSR technique. The whole process, including rapid template preparation (20-min), PSR amplification (60-min), ATSU treatment (5-min), and result reporting (within 2-min), can be finished within 90-min.

One original research article (Chiu and Yang) relates a high-sensitivity detection of the lung cancer biomarker CYFRA21-1 in serum samples using a carboxyl-MoS_2_ functional film for SPR-based immunosensors. The experiment succeeded in MoS_2_ reacted with chloroacetic acid giving carboxyl-MoS_2_ as the reaction product. The carboxyl-MoS_2_-based chip had a high affinity, with an SPR angle shift enhanced by 2.6-fold and affinity binding KA enhanced by 15-fold compared to a traditional SPR sensor. The results revealed that the carboxyl-MoS_2_-based chip had high sensitivity, specificity, and SPR signal affinity, while the CYFRA21-1 assay in spiked clinical serum showed a lower detection limit of 0.05 pg/mL. The carboxyl-MoS_2_-based chip detection value was about 104 times more sensitive than the limit of detection of an enzyme-linked immunosorbent assay (ELISA) (0.60 ng/mL). The results showed that the carboxyl-MoS_2_-based chip had the potential to rapidly assay complex samples including bodily fluids, whole blood, serum, plasma, urine, and saliva in SPR-based immunosensors to diagnose diseases including cancer.

Another original study (Bettini et al.) presents the supramolecular chiral discrimination of D-phenylalanine amino acid based on a perylene bisimide derivative. In the study, the interaction between homochiral substituted perylene bisimide molecule and the D enantiomer of phenylalanine amino acid was monitored. Spectroscopic transitions of PBI derivative in aqueous solution in the visible range were used to evaluate the presence of D-phenylalanine. UV-visible, fluorescence, FT-IR, and AFM characterizations showed that D-phenylalanine induces significant variations in the chiral perylene derivative aggregation state and the mechanism is enantioselective as a consequence of the 3D analyte structure. The interaction mechanism was further investigated in presence of interfering amino acid (D-serine and D-histidine) confirming that both chemical structure and its 3D structure play a crucial role for the amino acid discrimination. A D-phenylalanine fluorescence sensor based on perylene was proposed. A limit of detection (LOD) of 64.2 ± 0.38 nM was calculated in the range 10^−7^−10^−5^ M and of 1.53 ± 0.89 μM was obtained in the range 10^−5^ and 10^−3^ M.

The different approaches compiled in this Research Topic, pointed out the latest subjects and future challenges in this extensive area of research. We expect that the reader will find in this Research Topic valuable information regarding the state of the art, future trends and perspectives in theoretical and applicative bioanalysis.

## Author Contributions

All authors listed have made a substantial, direct and intellectual contribution to the work, and approved it for publication.

## Conflict of Interest

The authors declare that the research was conducted in the absence of any commercial or financial relationships that could be construed as a potential conflict of interest.

